# The decisive role of free water in determining homogenous ice nucleation behavior of aqueous solutions

**DOI:** 10.1038/srep26831

**Published:** 2016-05-26

**Authors:** Qiang Wang, Lishan Zhao, Chenxi Li, Zexian Cao

**Affiliations:** 1 Beijing National Laboratory for Condensed Matter Physics, Institute of Physics, Chinese Academy of Sciences, Beijing 100190, China; 2 Department of Physics, University of Science and Technology Beijing, Beijing 100083, China

## Abstract

It is a challenging issue to quantitatively characterize how the solute and pressure affect the homogeneous ice nucleation in a supercooled solution. By measuring the glass transition behavior of solutions, a universal feature of water-content dependence of glass transition temperature is recognized, which can be used to quantify hydration water in solutions. The amount of free water can then be determined for water-rich solutions, whose mass fraction, *X*_f_, is found to serve as a universal relevant parameter for characterizing the homogeneous ice nucleation temperature, the meting temperature of primary ice, and even the water activity of solutions of electrolytes and smaller organic molecules. Moreover, the effects of hydrated solute and pressure on ice nucleation is comparable, and the pressure, when properly scaled, can be incorporated into the universal parameter *X*_f_. These results help establish the decisive role of free water in determining ice nucleation and other relevant properties of aqueous solutions.

The microscopic mechanism of homogenous ice nucleation in pure water and aqueous solutions has been intensively studied over the past few decades. Although no consensus has been reached yet, the development of solute-free water domains of tetrahedrally coordinated structure is still regarded as the decisive stage of ice nucleation in solution^1^,^2^,^3^,^4^,^5^. Within such nanosized pure-water domains, a metastable intermediate phase, also called precursor of nucleus, emerges initially with local tetragonal symmetry^6^,^7^ and then grows into ice nucleus composed of randomly stacked layers of cubic and hexagonal sequences^7^,^8^. The presence of this precursory phase effectively reduces the nucleus/liquid interfacial tension, thus facilitates the occurrence of ice nucleation. A decrease in the probability of forming such precursory phase can explain the suppressing effect of solutes on ice nucleation in solutions^5^.

On the other hand, the relationship between the macroscopic features of solutions and the homogeneous ice nucleation temperature, *T*_H_, has also been investigated without considering the microscopic structural details of both the solution and ice nucleus therein. A prominent example is the semi-empirical water activity (*a*_w_) approach^9^,^10^,^11^,^12^. This thermodynamic approach stresses that *T*_H_ is uniquely related to *a*_w_, irrespective of the chemical nature of the solutes. This liquid-only criterion for the onset of freezing and the classical nucleation theory were suggested to be reconciled if the activation energy for water molecules to cross the nucleus/liquid interface is taken as a function of *a*_w_^9^. Later, a physical basis for this approach was proposed: for pure water under pressure *p* ≤ 1 kbar, *T*_H_ is nearly coincident with the temperature at which the compressibility of water reaches its maximum value, denoted herein as *T*(*κ*_max_)^10^. Different solutions of the same *a*_w_ have the same osmotic pressure, therefore, the same *T*(*κ*_max_) and *T*_H_. When cooled down to *T*(*κ*_max_), density or hydrogen boning network fluctuations becomes enough for forming critical-sized low density and tetrahedrally coordinated water domains needed by the formation of ice nucleus.

However, up to now, it still lacks a microscopic understanding of the effect of solute on homogeneous ice nucleation, for instance, how to estimate the effect of solute hydration on ice nucleation. Solute hydration plays an important role in determining many basic thermodynamic and kinetic properties of both solute and solvent in aqueous solutions. For describing *a*_w_, hydration water was suggested to be removed from solvent in calculating the effective concentration of solute^13^,^14^,^15^,^16^. But unsatisfactorily, in so doing the hydration number, *n*_h_, has only been simply adopted as an adjustable parameter without rigorous definition or justification^13^,^17^. To date, this problem still keeps unresolved because hydration water has been defined differently according to different properties of water involved, and *n*_h_ has been measured by various experimental techniques, including X-ray and neutron diffraction^17^,^18^, X-ray adsorption^19^, differential scanning calorimetric (DSC) measurement^20^, as well as Raman^21^, nuclear magnetic relaxation^22^, terahertz dielectric relaxation^23^, and infrared spectroscopies^23^,^24^. A strict definition of hydration water from the point of view of suppressing ice nucleation and an accurate quantification of *n*_h_ will open a possibility of discussing the effect of solute hydration on *T*_H_ and even *a*_w_, respectively, which will also help us microscopically understand the relation between *a*_w_ and *T*_H_.

When cooled solutions at moderate rates, water molecules in concentrated solutions can totally vitrify^25^,^26^,^27^,^28^, and those in water-rich solutions partially crystallize into primary ice and partially vitrify together with the solutes. These vitrified water molecules in low temperature phase of water-rich solutions are herein defined as hydration water. The value of *n*_h_ was quantified through comparing the glass transition temperature, *T*_g_, of hydration water with those at which concentrated solutions vitrify totally. Very interestingly, we observed that the mass fraction of free water, *X*_f_, can serve as a universal parameter for describing *T*_H_, *T*_m_, and even *a*_w_ of solutions of electrolytes and small organic molecules. This observation verified that the hydration water defined herein is rarely involved in the formation of ice nucleus, and contributes negligibly to *a*_w_, at the same time, stressed the key effect of amount of free water on the formability of ice nucleus and on the thermodynamic property of solutions. Moreover, we observed that, in depressing *T*_H_ and *T*_m_, pressure is equivalent to the mass fraction of hydrated solute, and also established the quantitative relationship between them.

## Results

### Quantification of hydration water

**Figure 1:**
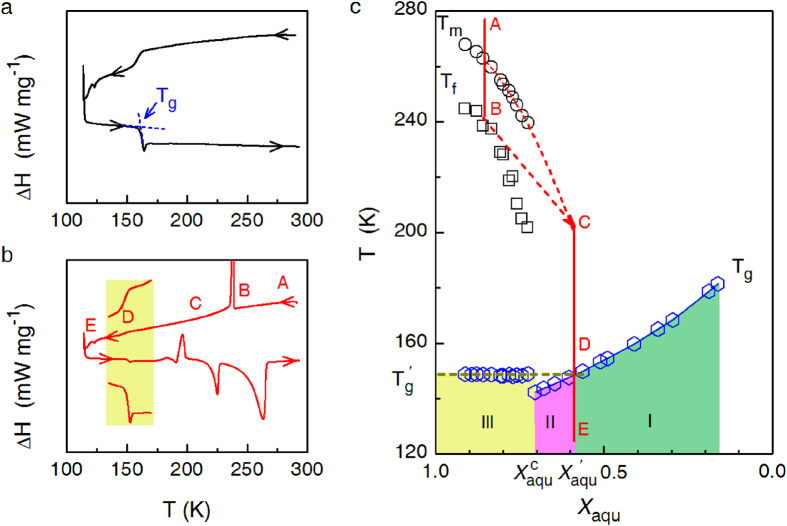
DSC thermograms of aqueous H_2_SO_4_ + HNO_3_ solutions with a mass fraction of water of (**a**) *X*_aqu_ = 0.39 and (**b**) *X*_aqu_ = 0.85. 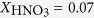 in both cases. (**c**) State diagram of H_2_SO_4_ + HNO_3_ solution. For solutions with 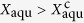 (zone III), primary ice precipitates first and then the residual freeze-concentrated solution vitrifies, obviously, at an almost constant temperature, termed 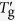. *T*_m_ and *T*_f_ refer to the melting and freezing points of primary ice precipitated within zone III. As shown in (**b**) and (**c**), during cooling solution with *X*_aqu_ = 0.85 from temperature point A, precipitation of primary ice begins at temperature point B and terminates at temperature point C. Below C, water content in freeze-concentrated solutions keeps nearly constant, which is equal to 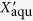.

We find that the water content dependence of *T*_g_ for aqueous solutions displays a quite universal feature, which in turn can provide a simple and reliable method for quantifying hydration number of solutes. Figure 1a,b show the DSC thermograms for the aqueous H_2_SO_4_ + HNO_3_ solutions with a mass fraction of water of *X*_aqu_ = 0.39 and 0.85, respectively. The mass fraction of HNO_3_ is fixed at *X*_HN*o*3_ = 0.07. The concentrated H_2_SO_4_ + HNO_3_ solutions such as the one with *X*_aqu_ = 0.39 can totally vitrify. This behavior was observed in solutions with increasing water content up to a critical value of 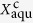 = 0.69 (Fig. 1c). For water-rich solutions with *X*_aqu_ > 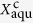, as illustrated by the sample with *X*_aqu_ = 0.85 in Fig. 1b, crystallization of primary ice occurs firstly, followed by the vitrification of freeze-concentrated solutions.

Those water molecules in vitrified freeze-concentrated solutions are defined as hydration water herein. The corresponding *n*_h_ can be deduced from the concentration of the freeze-concentrated solution. Traditionally, this concentration is determined by the point of intersection of *T*_g_ curve of concentrated solutions and the extrapolated *T*_m_ curve of ice in water-rich solutions. This method is valid mainly for solutions of larger organic molecules which have narrow temperature and/or concentration gaps between *T*_g_ and non-extrapolated *T*_m_ curves^20^,^26^. However, the error from extrapolating *T*_m_ curve cannot be neglected for solutions of electrolytes and small organic molecules, which have lager temperature and concentration gaps between *T*_g_ and non-extrapolated *T*_m_ curves. Moreover, it may be unreasonable to extrapolating *T*_m_ curve down to *T*_g_ curve. As can be seen from Fig. 1b,c, when cooled H_2_SO_4_ + HNO_3_ solution of *X*_aqu_ = 0.85 from temperature A, crystallization of primary ice begins at temperature B and finishes at C. Therefore, the concentration of freeze-concentrated solutions keeps constant when further decreasing temperature below C. This problem has already been noticed^28^, however, cannot be resolved mainly due to the difficulty in determining C point.

A comparison between *T*_g_ of freeze-concentrated solutions and those at which concentrated solutions totally vitrify can accurately determine the concentration of freeze-concentrated solutions. As shown in Fig. 1c, freeze-concentrated solutions vitrify at a nearly constant temperature, denoted as 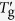. Therefore, the concentration of the freeze-concentrated phases corresponds to that of the solution that totally vitrifies at *T*_g_ = 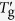 determined on the monotonous part of the *T*_g_ versus *X*_aqu_ curve. The determined concentration is denoted as 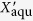, at which solution can also be written as M·*n*_h_ H_2_O, here M stands for the solute. With the known 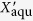 and 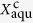, the solutions for a given solute can be categorized into three distinct zones with regard to different vitrification and crystallization behaviors of water (Fig. 1c). For solutions in zone I there is less water to complete the hydration; while solutions in zone III are water-rich, wherein ice precipitation occurs spontaneously in the cooling process. For solutions falling in zone II with 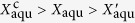, water can totally vitrify when cooled at moderate rates, however, crystallization of water can still be observed on the reheating process or after performing long-time holding treatment at temperatures above *T*_g_^29^. These recrystallized water molecules are normally named the freezable bound water.

The water-content dependence of *T*_g_ depicted in Fig. 1c was also measured in solutions of electrolytes such as LiCl, CaCl_2_, Ca(NO_3_)_2_, MnCl_2_, Mn(NO_3_)_2_, MgCl_2_, Mg(NO_3_)_2_, Mg(CH_3_COO)_2_, ZnCl_2_, FeCl_3_, Fe(NO_3_)_3_, CrCl_3_, Cr(NO_3_)_3_, AlCl_3_, HNO_3_-H_2_SO_4_, and of simple organic molecules such as glycerol, ethylene glycol (EG), polyethylene glycol (PEG) 300, dimethyl sulfoxide (DMSO), and 1,2,4-butanetriol; and some mixtures such as those of MgCl_2_ + ZnCl_2_, ZnCl_2_ + glycerol (see Supplementary Figs. 1–10). Experimental results show that the feature of *X*_aqu_-dependent *T*_g_ illustrated in Fig. 1c is quite universal at least to the systems investigated and cited in this work. The obtained 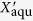 and *n*_h_ for the measured solutes are listed in Supplementary Table I.

### Mass fraction of free water as pertinent parameter

For dilute solutions in zones II & III with a *X*_aqu_ > 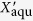, which containing more water than hydration, the mass fraction of free water, *X*_f_, can be calculated without any assumption. Briefly, for 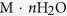, *X*_f_ is defined by


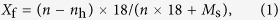


where *M*_s_ is the molar weight of the solute. For any aqueous solutions specified by a 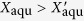, *X*_f_ can be directly calculated from


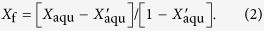


**Figure 2:**
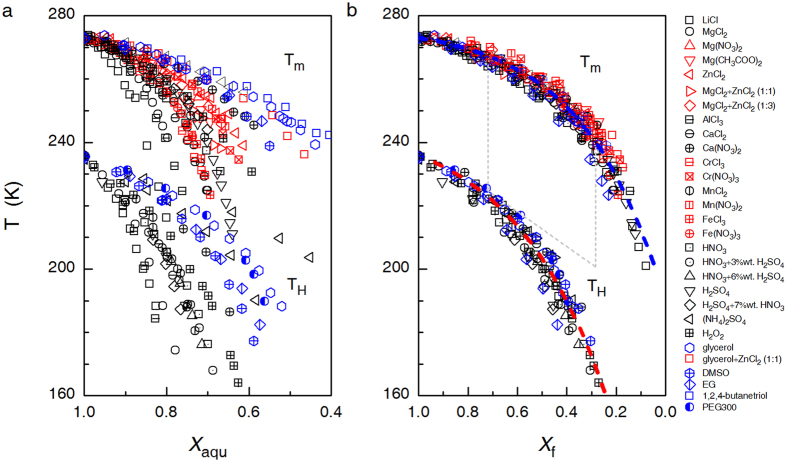
The homogeneous ice nucleation temperature, *T*_H_, and the melting temperature of ice, *T*_m_, as a function of (**a**) mass fraction of water, *X*_aqu_, and (**b**) mass fraction of free water, *X*_f_, for various aqueous solutions. For mixed solutions, the composition is specified either with the molar ratio of the solutes or the weight percentage of the minor solute. In (**b**), the dashed lines are added to emphasize the widen gap between *T*_H_ and*T*_m_ with increasing *X*_f_.

Remarkably, the original scattering *T*_H_ and *T*_m_ data when plotted against *X*_aqu_ (Fig. 2a) merge into two quite compact curves when plotted as a function of *X*_f_ (Fig. 2b), which can be well fitted by the functions 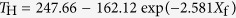 and 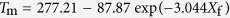, respectively. In Fig. 2, all the *T*_H_ data are cited from literatures published between 1980 and 2011, which were obtained by measuring emulsified samples with a diameter of 1~10 μm^11^,^26^,^30^,^31^,^32^,^33^,^34^,^35^,^36^,^37^,^38^,^39^. The *T*_m_ data were either extracted from the liquidus lines in the reported phase diagrams^25^,^26^,^28^,^34^,^39^,^40^,^41^,^42^ or read from the position of the endothermal peaks on DSC curves measured in the current work. We measured *T*_m_ data for solutions of Mg(CH_3_COO)_2_, EG, 1,2,4-butanetriol, glycerol + ZnCl_2_(1:1), MgCl_2_ + ZnCl_2_(1:1), MgCl_2_ + ZnCl_2_(1:3), and HNO_3_ + H_2_SO_4_. Additionally, the observed roughly universal *X*_f_ -dependence of *T*_H_ and *T*_m_ is also valid for electrolytic solutions wherein the freeze-concentrated phase preferably crystallizes instead of undergoing vitrification, such as aqueous solutions of H_2_O_2_, (NH_4_)_2_SO_4_, H_2_SO_4_, and HNO_3_ (see Supplementary Table 1 and the note therein). In this case, free water was still defined as the part of water that crystallizes into primary ice.

Figure 2b indicates that *T*_H_ depends solely on *X*_f_ and is insensitive to the specific solute-water interaction. This insensitiveness can be attributed to the screening effect of hydration water to the solutes. In other words, the influence of solutes on ice nucleation can be evaluated via the hydration capability of the solute, i.e., *n*_h_, which increases roughly linearly with the Gibbs energies of hydration of cations and molecules (see Supplementary Fig. 11), and increases from 6 for LiCl to about 19 for AlCl_3_ (Supplementary Table I). As we know, the number of water in the first and second hydration shells of ions is about 6 and 9~20 (depending on the type of cations), respectively^17^,^18^. Therefore, regarding influence upon ice nucleation occurring in dilute solutions, the solute−water interaction cannot extend beyond the first hydration shell for monovalent electrolytes and simple organic molecules, and cannot extend beyond the second hydration shell for multivalent electrolytes.

Strictly speaking, *n*_h_ can be regarded as a reflection of the ability of solutes to increase the fraction of high-density local heterogeneities in supercooled water. H.E. Stanley *et al*. made a similar suggestion for the effect of pressure on water structure^43^. Moreover, the gap between *T*_H_ and *T*_m_ curves plotted in Fig. 2b becomes widen with decreasing *X*_f_ (or with increasing the mass fraction of hydrated solute (1 − *X*_f_)). This behavior is also analogous to the effect of pressure^44^. Therefore, the following text discusses the equivalent relationship between pressure and (1 − *X*_f_) from the point of view of depressing *T*_H_.

The equivalency relation between solute concentration, *c*, and external pressure, *P*, with regard to their effect upon reducing *T*_H_ has drawn much attention, and such an equivalency relation was first observed in alkali halide solutions^45^. However, no valuable information has ever extracted because the variation of *T*_H_ with *c* is apparently very sensitive to the solute type. Koop *et al*. also analyzed this problem on the basis of the concept of *a*_w_^9^. They found that *T*_H_(*c*, *P*) = *T*_H_(*c*^eff^, *P* = 0), where *c*^eff^ refers to an effective solute concentration, which is defined via the equation 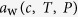 = 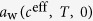.

**Figure 3:**
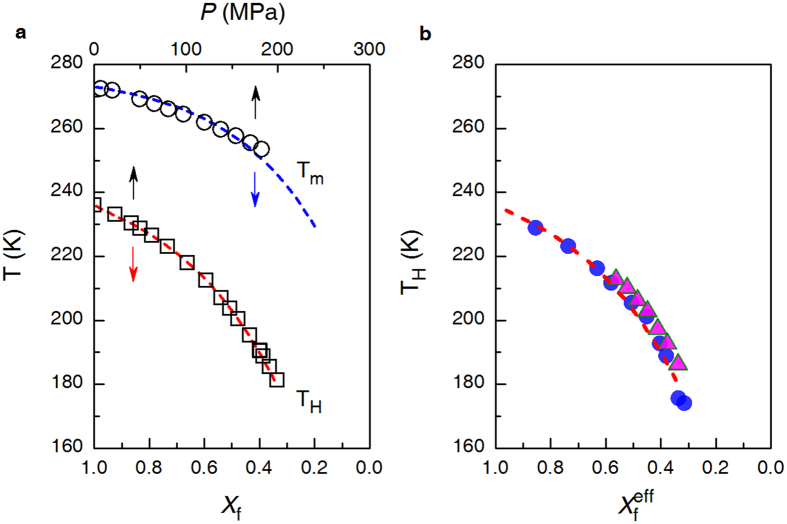
(**a**) The homogeneous ice nucleation temperature, *T*_H_, as a function of pressure, *P*, for pure water (black square), and of the mass fraction of free water, *X*_f_, fitted for solutions referring to Fig. 2b (red dashed line). For comparison, melting temperature ice, *T*_m_, as a function of pressure for pure water (black circle), and of *X*_f_ for solutions (blue dashed line) were also plotted. (**b**) *T*_H_ as a function of the effective mass fraction of free water, 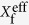, for pure water under variable pressures and solutions under atmospheric pressure (red dashed line), and for 5.56 mol. kg^−1^ glycerol solutions under pressure up to about 120 MPa (triangle) and for 1.0 mol. kg^−1^ LiCl solution under pressure up to about 190 MPa (circle).

Figure 3a comparatively plots the *X*_f_–dependence of *T*_H_ for solutions and *P*–dependence of *T*_H_ for pure water^45^. Obviously, in depressing *T*_H_, *P* can be linearly scaled against the mass fraction of hydrated solute in the following way:


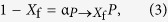


where *P* is in MPa, and the coefficient *α*_*P*→*X*f_ = 3.32×10^−3 ^MPa^−1^. This linear scaling relation in Eq. (3) holds for pressure range 0.1 < *P* < 200 MPa, corresponding to 180 K < *T*_H_ < 236 K. This is to say that *T*_H_ values are approximately the same for pure water under *P* and solutions with a mass fraction of free water 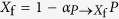.

The dominating mechanism for solutes to suppress ice nucleation is to combine with hydration water so as to reduce the amount of free water. We applied this picture to understand the effect of pressure. For a solution of *X*_f_, when further subject to an external pressure of *P*, an effective mass fraction of free water, 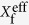, can be introduced in analog to the definition for *X*_f_,


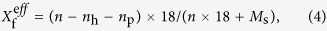


where *n*_p_ specifies the effect of the applied pressure on the part of free water denoted by (n − *n*_h_), as if it further reduced the amount of free water as by hydration. By virtue of Eq.(3), it should have


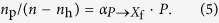


Thus we arrive at the formula.


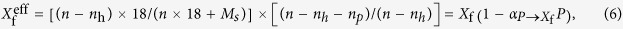


where *X*_f_ and 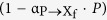 represent the contributions from solutes and pressure, respectively. Eq.(6) implies that in reducing the amount of free water in a solution, the pressure and the solute work in close collaboration. The hydrated solutes exert influence on the local hydrogen bonding network in water, while, pressure promotes the high-density non-tetrahedral local structure in free water, both lead to the suppression of ice nucleation probability.

Figure 3b shows the 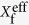 dependence of *T*_H_ for solutions of 1.0 mol. kg^−1^ LiCl^45^ and 5.56 mol. kg^−1^ glycerol^46^ under pressures up to 200 MPa (for NaCl, see Supplementary Fig. 12). As expected, all the *T*_H_ data points fall exclusively onto the same curve referring to that in Fig. 2b. The regular dependence of *T*_H_ obtained under various concentrations or pressures on the parameter 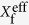 also suggests that the pressure in the given range has almost no bearing on the hydration water of LiCl and glycerol.

## Discussion

Interestingly, the *T*_H_ data referred to Fig. 2a also merge into a very compact distribution when plotted as a function of *a*_w_^9^, which is a good thermodynamic parameter for comprehensively describing the effect of solutes on ice nucleation. *a*_w_ and the related osmotic pressure have been widely used to characterize the formability of nanosized water domains in supercooled solutions^4^,^10^, or the change in entropy and the activation energy required for water molecules to cross the nucleus/liquid interface^9^,^12^. The application of *a*_w_ undoubtedly provides a simplified treatment of the complex interactions among the different components in a solution. However, this simplified treatment also hinders understanding the detailed role of solute in determining ice nucleation, in part because of a lack of general relations between *a*_w_ and other features of the solution concerning the solute-water interaction^15^.

**Figure 4:**
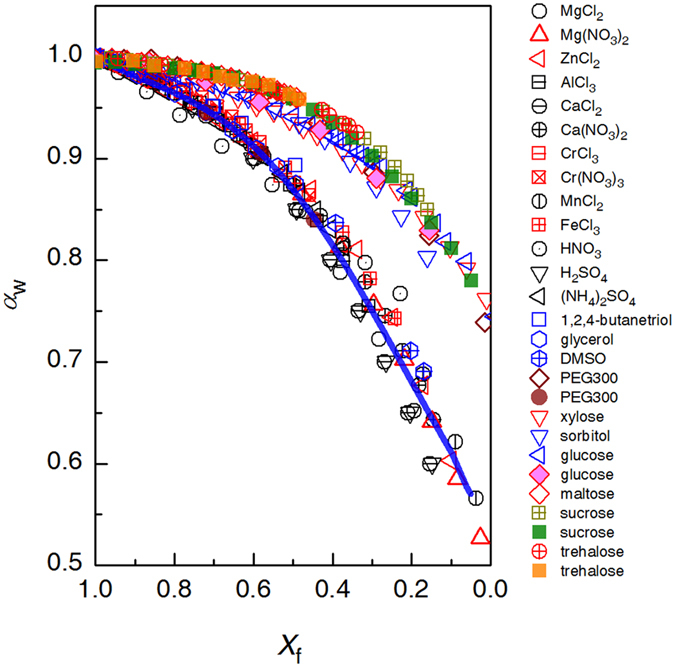
Water activity, *a*_w_, measured at 298 K for various aqueous solutions plotted against *X*_f_ (open symbol). Also *a*_w_ at *T*_m_ for solutions of PEG 300, glucose, sucrose, and trehalose molecules (solid symbol) are included for comparison. The blue solid line results from the calculated *a*_w_(*T*_m_) according to Eq. (7) in conjunction with the *T*_m_ ~ *X*_f_ relation referred to Fig. 2b.

Figure 4 plots *a*_w_ measured at 298 K for various aqueous solutions against *X*_f_. For comparison, Fig. 4 also plots *X*_f_ dependence of *a*_w_ at *T*_m_ for solutions calculated according to the following equation:


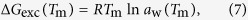


where 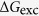 is the excess Gibbs free energy of supercooled water, which is defined as the excess heat capacity of supercooled bulk water with respect to bulk ice^47^, and *R* is the gas constant. The data points are no more randomly scattered as plotted versus molar fraction of water or even versus molar fraction of free water (Supplementary Figs 13 and 14), rather they fall onto two seemingly distinct branches (Fig. 4). The lower branch includes electrolytic solutions and the solutions of small organic molecules such as 1,2,4-butanetrio, glycerol, and DMSO, all measured at 298 K^13^,^47^,^48^,^49^,^50^,^51^,^52^,^53^,^54^,^55^,^56^,^57^,^58^,^59^. Consequently, the relationship between *a*_w_(*T*_m_) ~ *X*_f_ can be deduced by relationship between *a*_w_(*T*_m_) and *T*_m_ established according to Eq. (7) and that between *T*_m_ and *X*_f_ plotted in Fig. 2b. Noticeably, the deduced *a*_w_(*T*_m_) as a function of *X*_f_ (blue line) fits well *a*_w_ data measured at 298 K for the electrolytic solutions and those of small organic molecules. The *a*_w_ data measured at 298 K differ from those measured at the corresponding *T*_m_ only negligibly, thus we suggest that *n*_h_ is insensitive to temperature.

The quite universal dependences of *T*_H_ and *a*_w_(*T*_m_) on *X*_f_, at least for electrolytic solutions, explains the successful application of *a*_w_ in describing the effect of solutes on ice nucleation, as *a*_w_ indirectly reflects the availability of free water in supercooled solutions.

The above-mentioned fitted *T*_H_ versus *X*_f_ and *T*_m_ versus *X*_f_ relations are not valid for solutions of large organic molecules illustrated in Fig. 4, including solutions of xylose, sorbitol, glucose, maltose, sucrose, and trehalose. Obviously these *a*_w_ data fall apart from those for electrolytes and small organic molecules, and yet, interestingly, they also roughly merge into one single curve. This difference in *a*_w_versus *X*_f_ between electrolytic solutions and those of large organic molecule may lie in the structural heterogeneity in the latter ones. Of the similar*X*_f_ levels, the region of free water may be larger due to the aggregation of (unevenly) hydrated large organic molecules, thus manifesting a higher value for *a*_w_ and *T*_H_. PEG 300 is an exception, that its data at *T*_m_ and at 298 K fall on two branches respectively, i.e., its hydration water number is sensitive to temperature^60^.

The *X*_f_ ~ *a*_w_ relation displaying two distinct branches reminds us that, for the same nominal *X*_f_, it refers to a larger *a*_w_ thus a higher *T*_H_ for solutions of large organic molecules (data not shown). As *T*_H_ depends on the local amount of free water in solution, this clearly results from the structural inhomogeneity in those solutions arising from aggregation of the large organic molecules. Thus one must be cautious in understanding *T*_H_, *T*_m_ and other water-related properties for such solutions, since the locally available amount of free water is not necessarily consistent with the nominal concentration of the solution. This case can partially explain the invalidity of colligative property for describing the effect of solutes on *T*_H_ and *a*_w_ even when hydration is also taken into consideration.

The universal dependence of *T*_H_ on *X*_f_, or *T*_H_(*c*, *P*) on 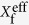, and even the deviation of *a*_w_(*T*_m_) ~ *X*_f_ for electrolytic solutions and solutions of larger organic molecules, lead to recognition of the pivotal role of free water, or the hydration water, in determining the ice nucleation behavior. The solute ions or molecules differ, with regard to suppressing the ice nucleation temperature, in their hydration capability. Ice nucleation in different solutions can thus be understood on the same footing with the concept of hydrated solute or mass fraction of free water. We suggest that hydration of solutes reflect their abilities to increase the fraction of high-density heterogeneities in supercooled water. Different solutions at the same mass fraction of free water have the same temperature dependence of probability of forming critical-sized low-density domain in supercooled free water. However, it still is difficult to estimate the size of the free water domains in solutions from the nominal water content of the solution, as the free water regions are interconnected, and at least in solutions of large organic molecules the distribution of free water may show a severe inhomogeneity. Moreover, a fact remains quite puzzling that why it is mass fraction of free water that universally describe the ice nucleation in solutions.

In summary, by measuring the water-content dependence of glass transition temperature for many different aqueous solutions we recognized that the nearly constant glass transition temperature for the freeze-concentrated phases from the super-cooled water-rich solutions provides a method to quantify the number of hydration water for a given solute. A relevant parameter, the mass fraction of free water, can be well defined and determined. A universal dependence on this parameter was established for the homogeneous ice nucleation temperature, *T*_H_, the melting temperature of primary ice, *T*_m_, and even *a*_w_(*T*_m_). This observation is also valid for water-rich solutions in which the freeze-concentrated phase preferably crystallizes instead of undergoing vitrification. When properly scaled, for suppressing ice nucleation, the effect of pressure can be incorporated into an effective mass fraction of free water. The one-to-one correspondence between water activity and mass fraction of free water (for electrolytic solutions to the least) explains the validity of water activity in describing the homogeneous ice nucleation behavior in supercooled solutions. It reveals the fact that water activity is essentially a measure of free water.

## Methods

### Samples

High-purity water was prepared by using a Millipore Milli-Q system. The solutes LiCl (anhydrous, 99.99%), CaCl_2_ (anhydrous, 99.99%), Ca(NO_3_)_2_·4H_2_O (ACS reagent, 99%), MgCl_2_ (anhydrous, 99.99%), MnCl_2_·4H_2_O (99.99%), ZnCl_2_ (anhydrous, 99.99%), CrCl_3_·6H_2_O (98.0%), AlCl_3_·6H_2_O (99%), FeCl_3_·6H_2_O (99%), HNO_3_ (70%, purified by re-distillation to 99.999%), glycerol (99.5%), ethylene glycol (anhydrous, 99.8%), dimethyl sulfoxide(anhydrous, 99.9%), polyethylene glycol 300 (BioUltra) were purchased from Sigma-Aldrich. Other solutes include H_2_O_2_ (50 wt.% in H_2_O, 85 wt.% in H_2_O, Beijing Chem. Tech. Co.) and H_2_SO_4_ (98%, Sinopharm Chemical Reagent).

### Thermal measurement

Differential scanning calorimetric measurement on droplets (∼5.0 μL) of aqueous solutions was performed on a calorimeter (PE DSC8000) operating at a cooling/heating rate of 20 K min.^−1^ unless otherwise specified. When cooled down to 110 K, the sample was held at this temperature for 1 min. before the heating procedure began. All of the DSC curves were normalized against sample weight.

## Additional Information

**How to cite this article**: Wang, Q. *et al*. The decisive role of free water in determining homogenous ice nucleation behavior of aqueous solutions. *Sci. Rep*. **6**, 26831; doi: 10.1038/srep26831 (2016).

## Supplementary Material

Supplementary Information
