# Supplementary material for: The decisive role of free water in determining homogenous ice nucleation behavior of aqueous solutions
Source: Sci Rep. 2016 May 26;6:26831. doi: 10.1038/srep26831 (PMC4881027; doi:10.1038/srep26831)
Supplement: Supplementary Information [file srep26831-s1.pdf]

# Supplementary Information

## The decisive role of free water in determining homogeneous ice nucleation behavior of aqueous solutions

Qiang Wang,[†] Lishan Zhao, [‡†] Chenxi Li,[†] and Zexian Cao[†]

[†]Beijing National Laboratory for Condensed Matter Physics, Institute of Physics, Chinese Academy of Sciences, Beijing 100190, China

[‡]Department of Physics, University of Science and Technology Beijing, Beijing 100083, China

This supplementary information includes:

a) Figures. S1-S14

b) Table S1 with explanatory notes

c) References

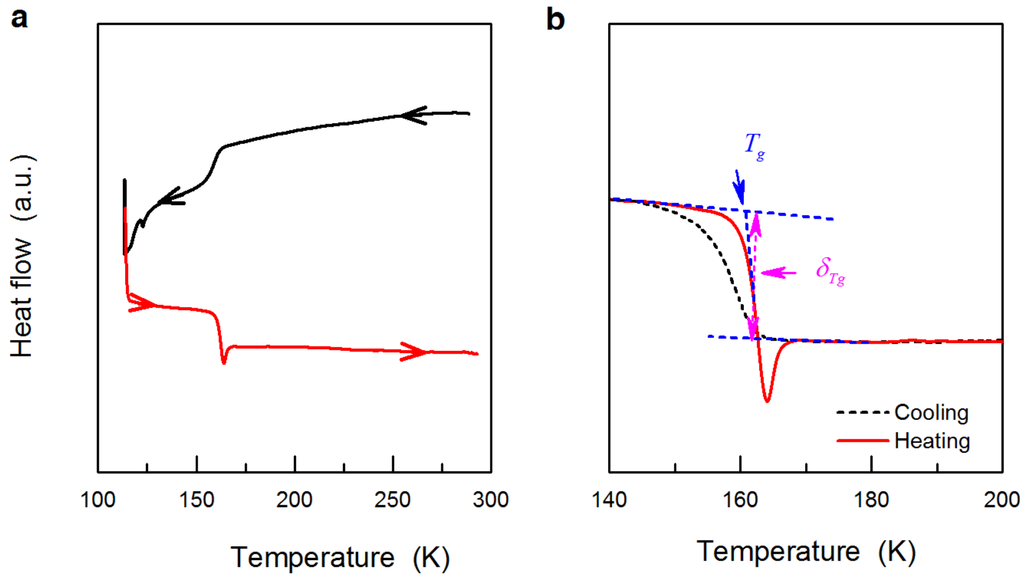


**Figure S1.** Extraction of the **o**nset temperature of glass transition, $T_\mathrm{g}$, and the heat flow change at $T_\mathrm{g}$, denoted as $\delta_{\mathrm{T_g}}$, from DSC heating curve. (**a**) Normalized DSC thermogram of $HNO_3$+$H_2SO_4$ solution with $X_{\mathrm{HNO_3}} + X_{\mathrm{H_2SO_4}}$= 0.65 wherein $X_{\mathrm{HNO_3}}$= 0.07; (**b**) Local magnification of the heat flow step corresponding to the glass transition.

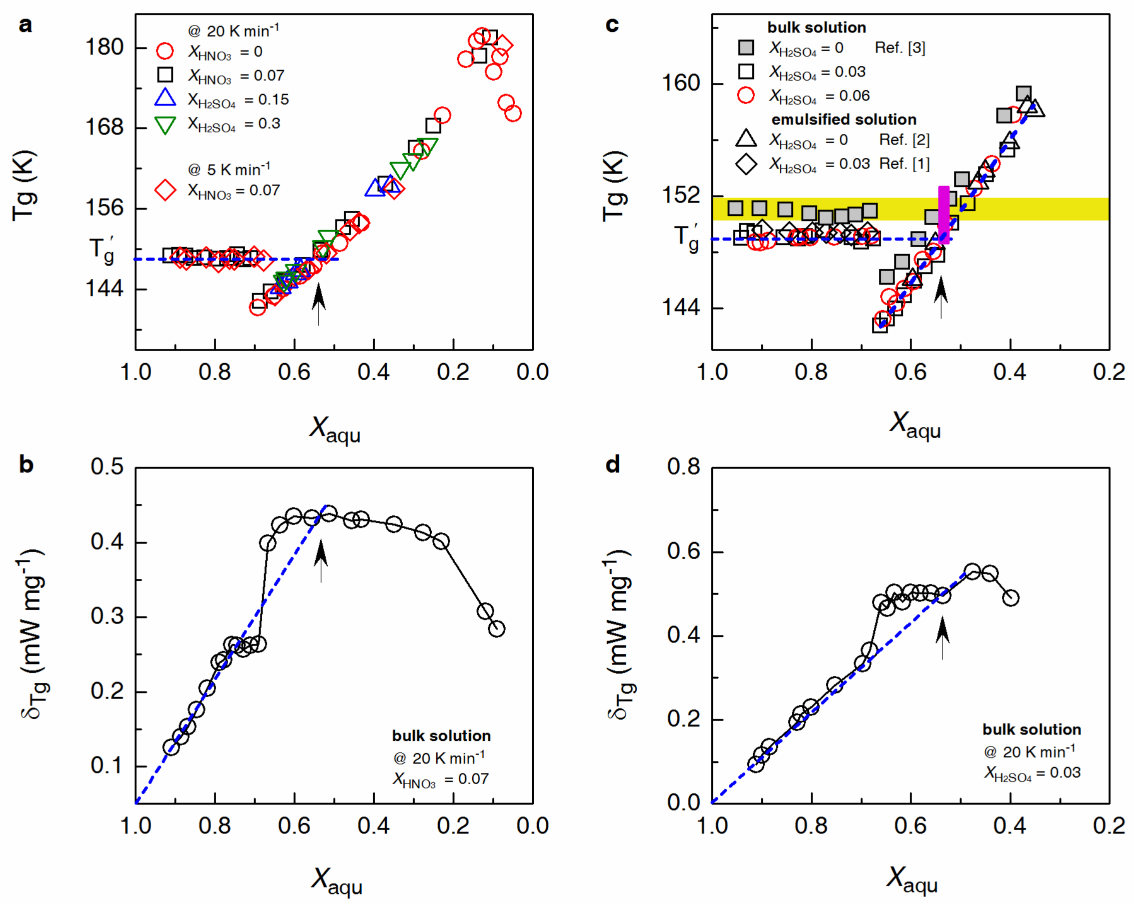


**Figure S2.** $T_g$ and $\delta_{T_g}$ versus mass fraction of water $X_{aqu}$ for aqueous $H_2SO_4$+$HNO_3$ solutions with a fixed $X_{HNO_3}$ (**a, b**) or a fixed $X_{H2SO4}$ (**c, d**). In (**c**), $T_g$ of bulk and emulsified $H_2SO_4$+$HNO_3$ solutions are presented for comparison. $X'_{aqu}$= 0.56 and 0.54 for $H_2SO_4$+$HNO_3$ solution with $X_{HNO_3}$= 0.07 and $X_{H_2SO_4}$=0.03, respectively. In (**b**) and (**d**), the blue dashed line is drawn from the equation $\delta_{T_g} = \delta'_{T_g} \cdot (1 - X_{aqu})/(1 - X'_{aqu})$, where $(1 - X_{aqu})/(1 - X'_{aqu})$ is the concentration of the freeze-concentrated solution calculated using the lever rule, $\delta'_{T_g}$ corresponds to $\delta_{T_g}$ of the solution with $X'_{aqu}$.

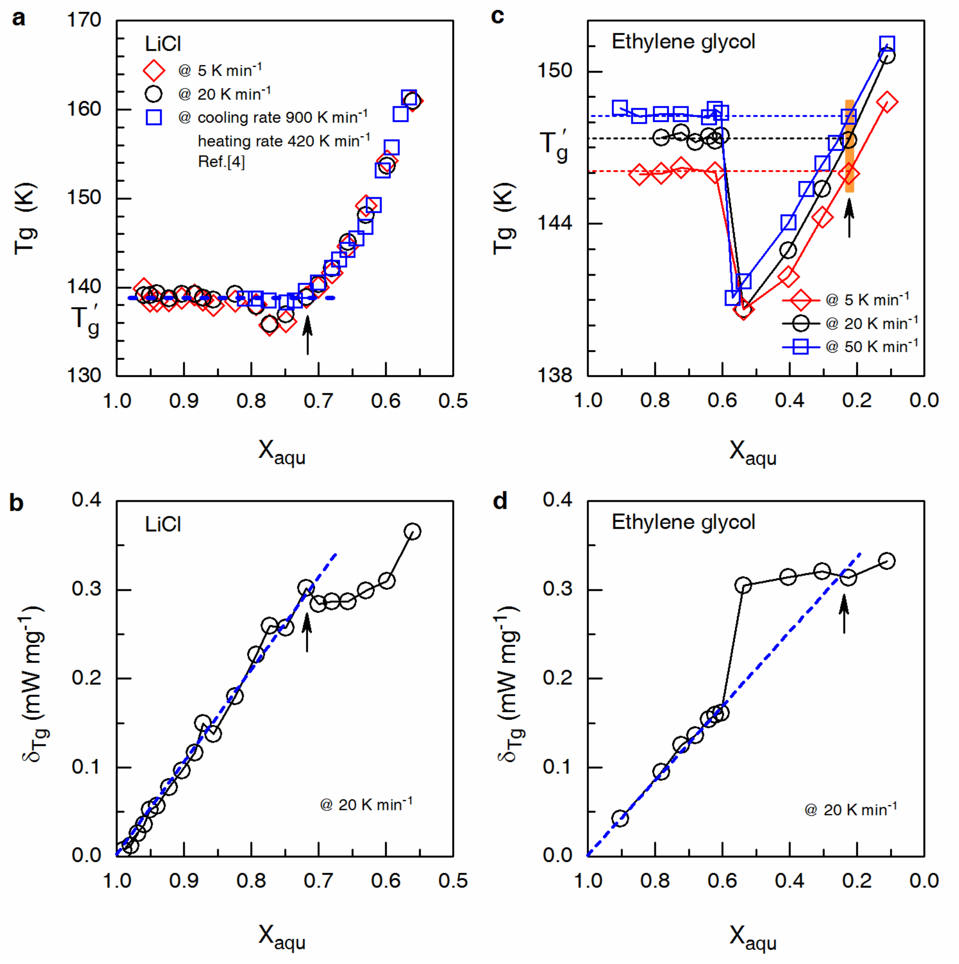


**Figure S3.** $T_g$ and $\delta_{T_g}$ versus $X_{aqu}$ for aqueous solutions of LiCl (**a, b**) and ethylene glycol (**c, d**). $T_g$ data extracted from Ref.[4] are also illustrated in (**a**) for comparison. $X'_{aqu}$= 0.72 and 0.24 for LiCl and ethylene glycol solutions, respectively.

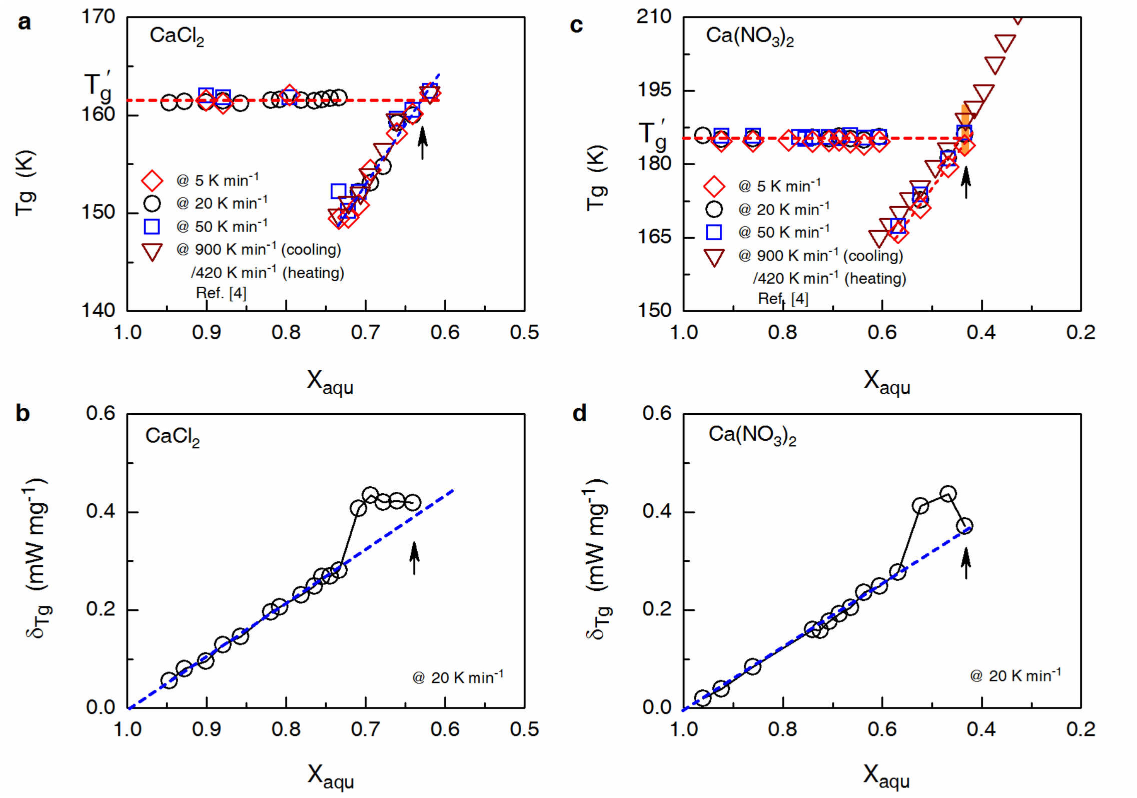


**Figure S4.** $T_g$ and $\delta_{T_g}$ versus $X_{aqu}$ for aqueous solutions of $CaCl_2$ (**a,b**) and $Ca(NO_3)_2$ (**c,d**). $T_g$ data extracted from Ref.[4] are also shown in (**a**) and (**c**) for comparison. $X'_{aqu}$= 0.62 and 0.43 for $CaCl_2$ and $Ca(NO_3)_2$ solutions, which correspond to the values of $CaCl_2$:$10H_2O$ and $Ca(NO_3)_2$:$7H_2O$, respectively. The same type of cation but different anions is shared by these two solutes. Although cations have a stronger interaction with water than anion, an obvious difference in hydration number can still be observed. This difference suggests that anions have a non-negligible effect on $X'_{aqu}$, probably due to a cooperativity in ion hydration[5]. At present, it is still difficult to understand how hydration water molecules are shared by cation and anion, as already stressed by Stoke and Robinson[6].

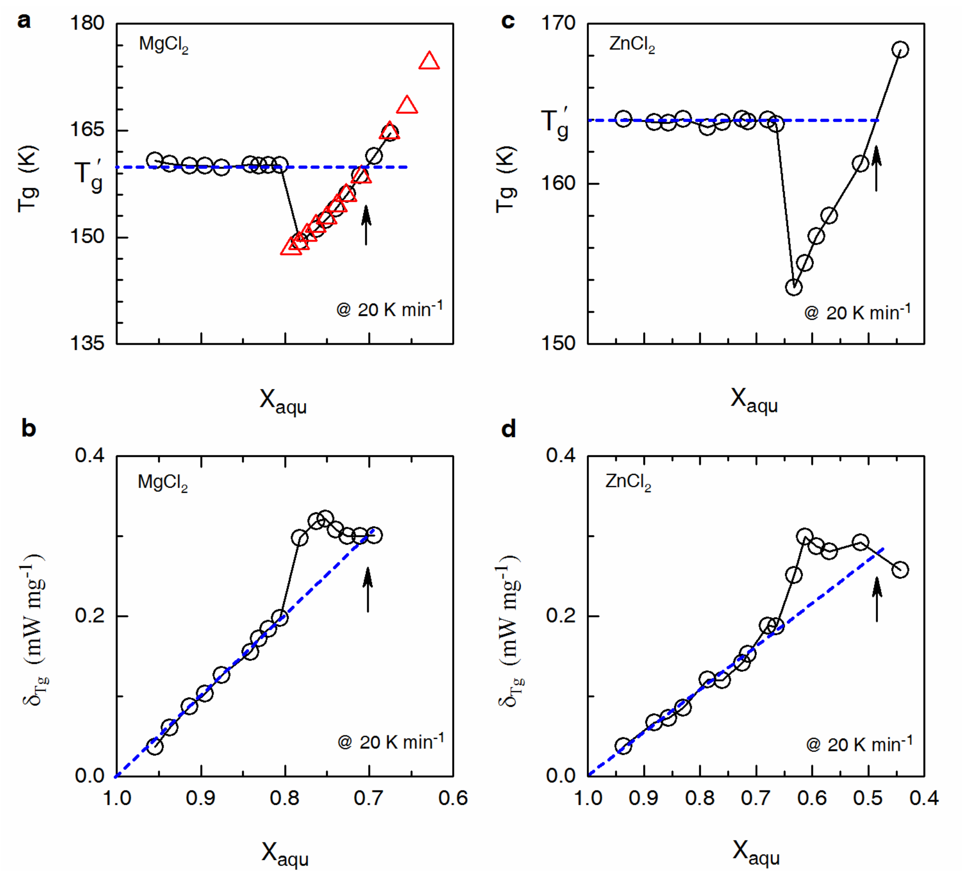


**Figure S5.** $T_g$ and $\delta_{T_g}$ versus $X_{aqu}$ for aqueous solutions of $MgCl_2$ (**a,b**) and $ZnCl_2$ (**c,d**). $T_g$ data extracted from Ref.[4] are also illustrated in (**a**) for comparison. $X'_{aqu}$= 0.70 and 0.48 for $MgCl_2$ and $ZnCl_2$ solutions, respectively.

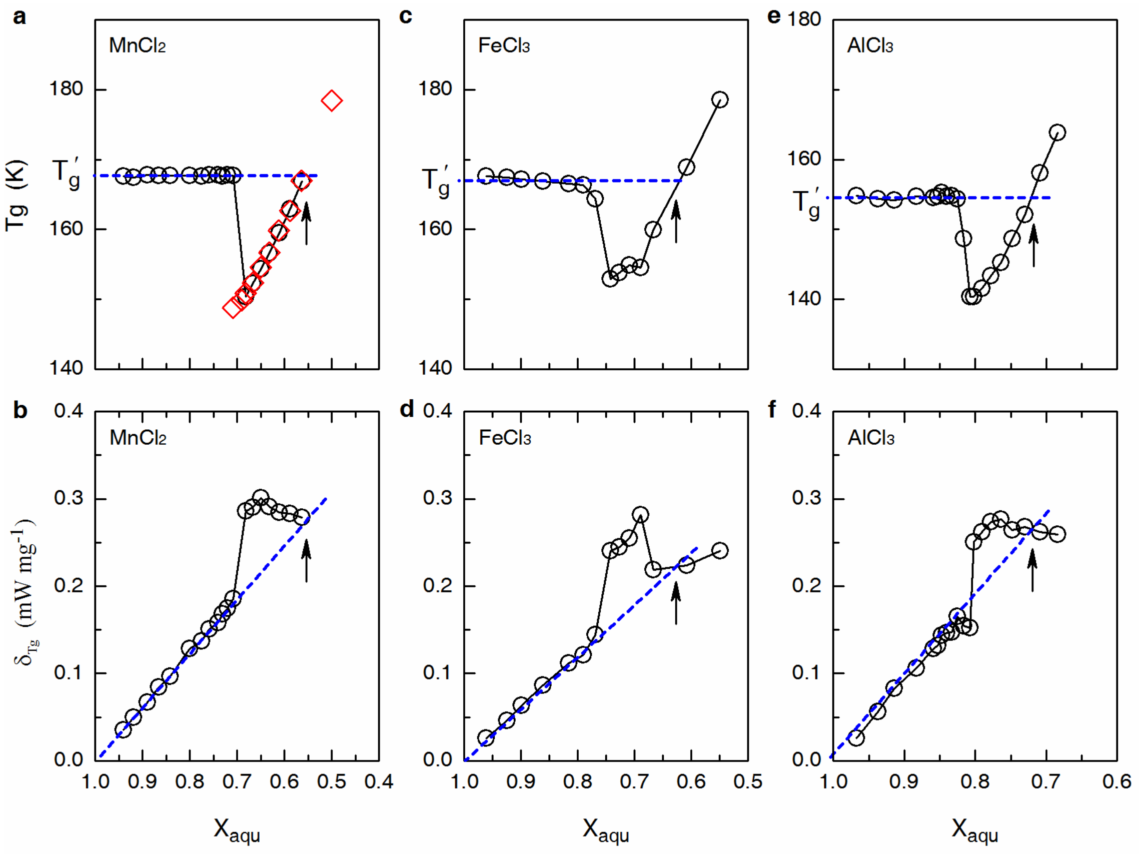


**Figure S6.** $T_g$ and $\delta_{T_g}$ versus $X_{aqu}$ for aqueous solutions of $MnCl_2$ (**a,b**), $FeCl_3$ (**c,d**), and $AlCl_3$ (**e,f**). $T_g$ data from Ref.[4] (red diamond) are also shown in (**a**) for comparison. $X'_{aqu}$= 0.56, 0.62, and 0.72 for solutions of $MnCl_2$, $FeCl_3$, and $AlCl_3$, respectively.

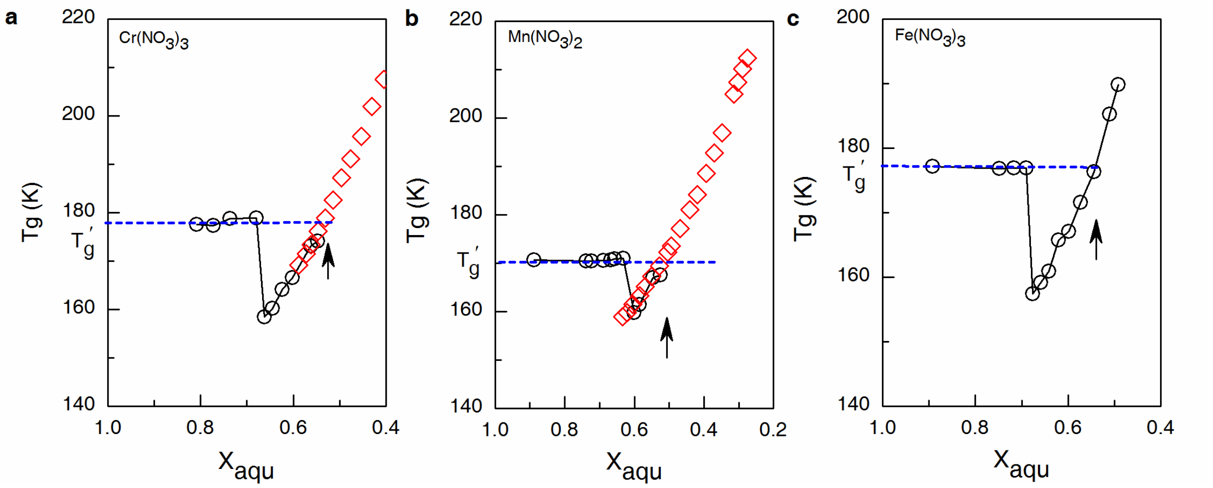


**Figure S7:** $T_g$ versus $X_{aqu}$ for aqueous solutions of $Cr(NO_3)_3$ (**a**), $Mn(NO_3)_2$ (**b**), and $Fe(NO_3)_3$ (**c**). $T_g$ data from Ref.[4] (red diamond) are also shown in (**a**) and (**b**) for comparison. $X'_{aqu}$= 0.54, 0.51, and 0.54 for solutions of $Cr(NO_3)_3$, $Mn(NO_3)_2$, and $Fe(NO_3)_3$, respectively.

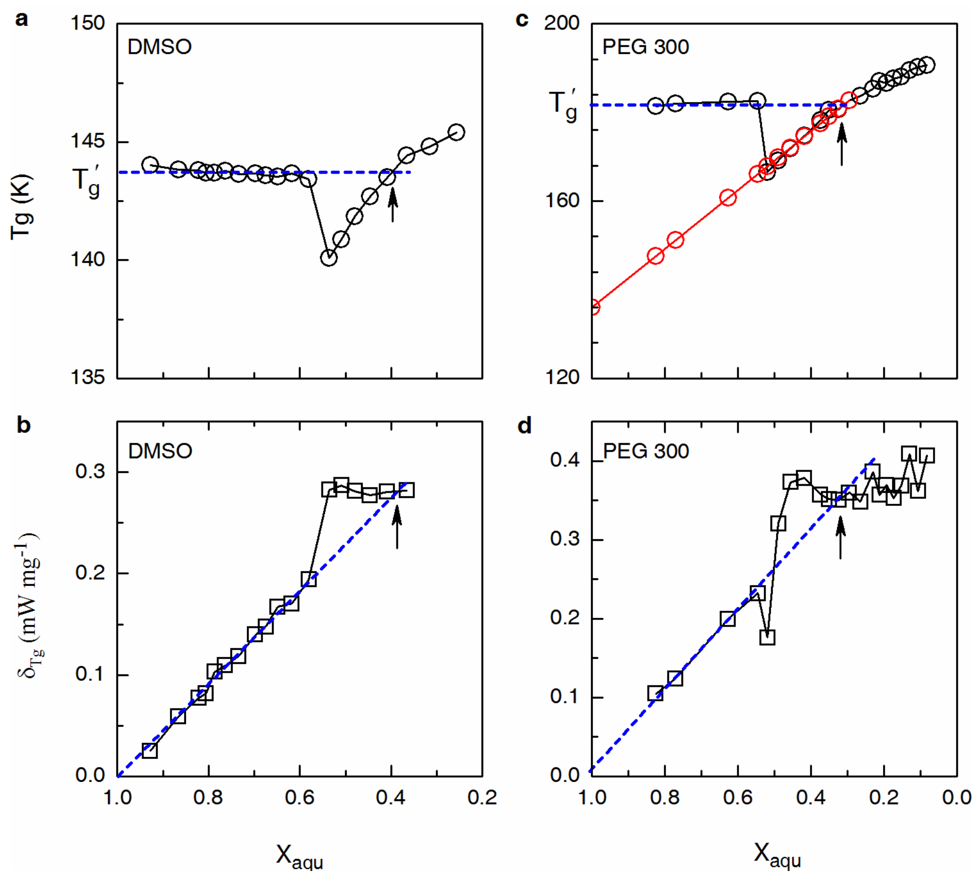


**Figure S8.** $T_g$ and $\delta_{T_g}$ versus $X_{aqu}$ for solutions of dimethyl sulfoxide (DMSO) (**a, b**) and PEG 300 (**c, d**). $X'_{aqu}$= 0.41 and 0.29 for DMSO and PEG 300 solutions, respectively. Red circles in (**c**) denote the fitting results according to the Gordon-Taylor equation, i.e., $T_g = (X_1 T_{g_1} + kX_2 T_{g_2})/(X_1 + kX_2)$, where $X_1$ and $X_2$ are the mass fractions of PEG300:6.8$H_2O$ and free water; $T_{g_1}$= 181.8 K and $T_{g_2}$= 136 K; $k$ is the Gordon-Taylor coefficient, here $k$ = 1.

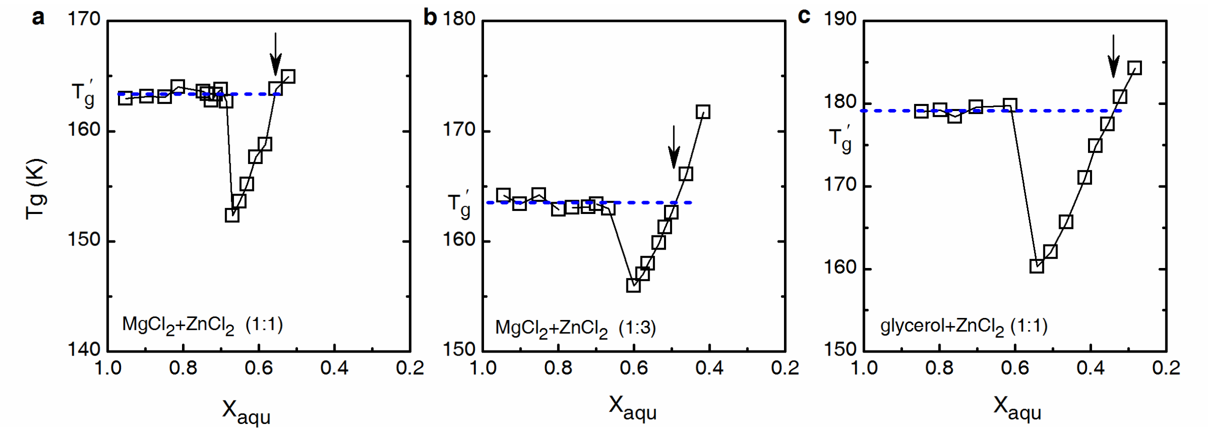


**Figure S9.** $T_g$ and $\delta_{T_g}$ versus $X_{aqu}$ for (**a**) $MgCl_2$+$ZnCl_2$ with a molar ratio of 1:1, **(b)** $MgCl_2$+$ZnCl_2$ with a molar ratio of 1:3, and **(c)** $ZnCl_2$+glycerol with a molar ratio of 1:1.

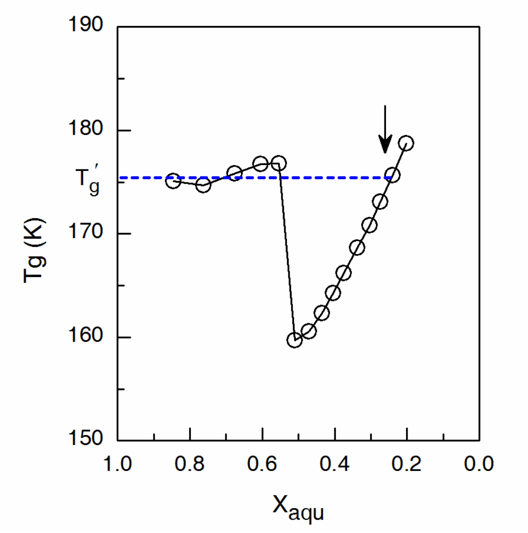


**Figure S10:** $T_g$ versus $X_{aqu}$ for solutions of 1,2,4-butanetriol. $X'_{aqu}$= 0.24.

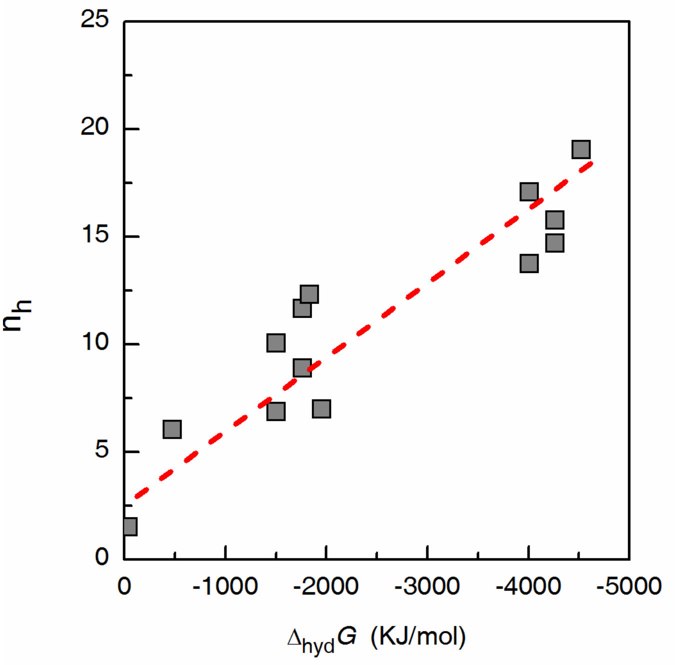


Figure S11: The dependence of hydration number on the Gibbs energies of hydration of cations or molecules.

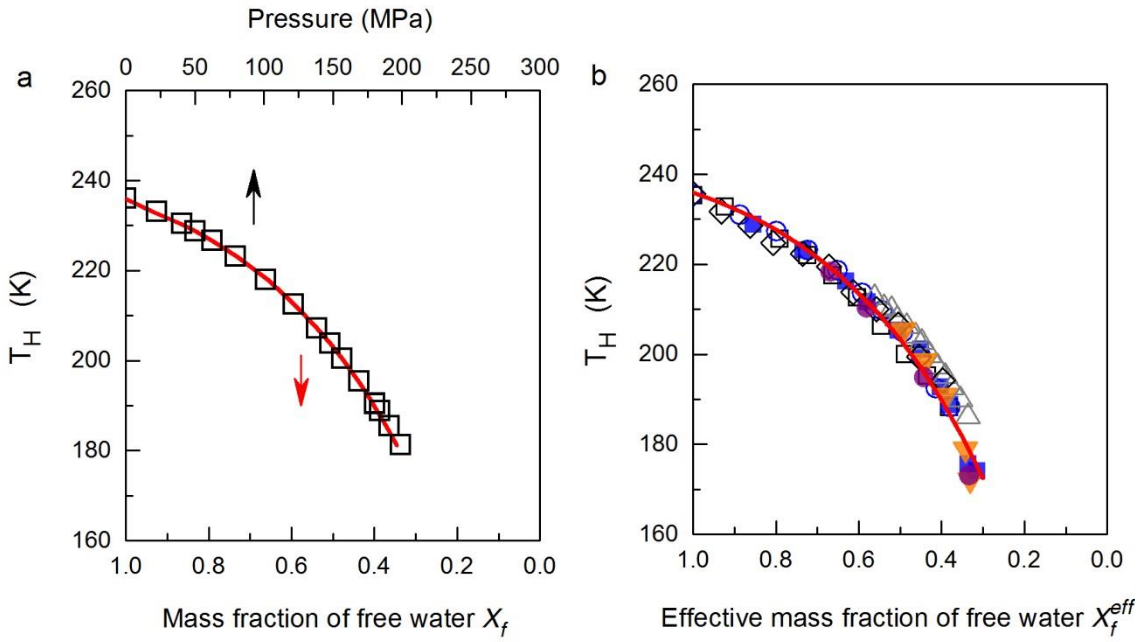


**Figure S12.** Equivalency of pressure and $X_\mathrm{f}$ in suppressing $T_\mathrm{H}$. (**a**) $T_\mathrm{H}$ of aqueous solutions at ambient pressure (red solid line) plotted against $X_\mathrm{f}$., and that of pure water at pressures up to about 200 MPa (hollow squares); (**b**) $T_\mathrm{H}$ of pure water (red solid line) and solutions (hollow and filled symbols) under different pressures plotted against $X_\mathrm{f}^\mathrm{eff}$. Solutions of various concentrations at ambient pressure: hollow square: LiCl; hollow circle: glycerol; hollow diamond**:** NaCl ($n_\mathrm{h} = 4.5$, see Table S1); Solutions at different pressures: filled square: 1 mol.·kg$^{-1}$ LiCl; hollow triangle: 5.56 mol.·kg$^{-1}$ glycerol; filled downward triangle: 4.63 mol.·kg$^{-1}$ NaCl, and filled circle: NaCl solutions with different concentrations but at a fixed pressure of 100 MPa.

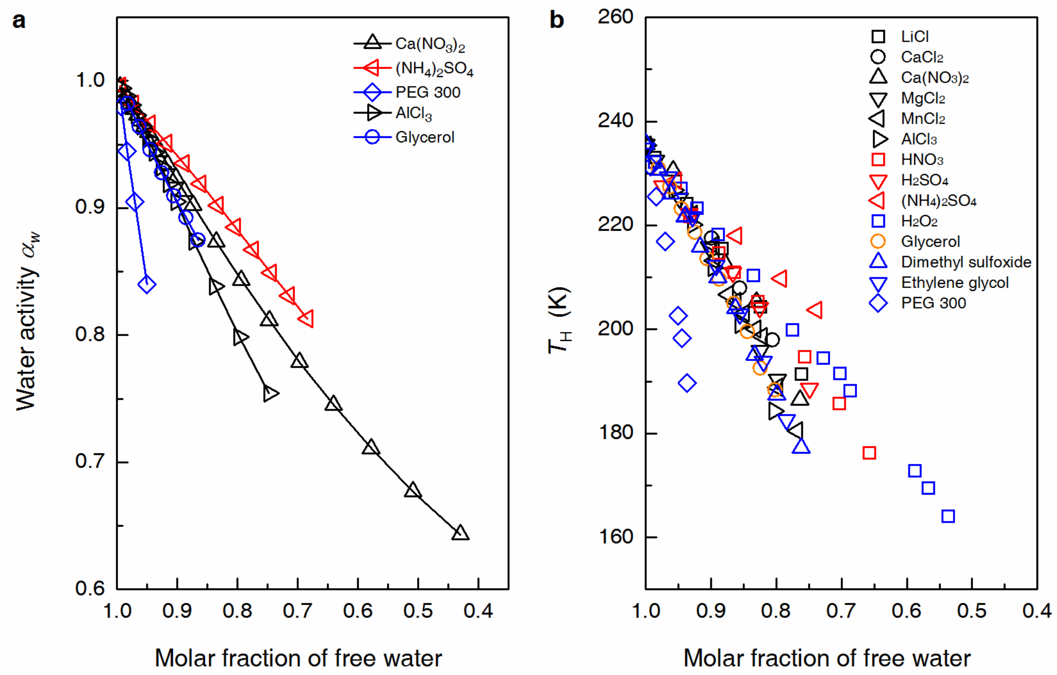


**Figure S13.** Water activity $a_{\mathrm{w}}$ (**a**) and homogeneous ice nucleation temperature $T_{\mathrm{H}}$ (**b**) versus molar fraction of free water in solution. The molar fraction of free water is given by $(n - n_{\mathrm{h}})/(n - n_{\mathrm{h}} + v)$, where $n$ is the molar ratio of water to solute, $n_{\mathrm{h}}$ is the molar ratio of hydration water to solute, $v$ is the van't Hoff index of the solute. In ideal case, $v$ = 2 for LiCl, 3 for $Ca(NO_3)_2$, and 4 for $AlCl_3$. Here, the cation or anion bound by hydration water is modeled as a single particle, and the hydration water is excluded when calculating the molar fraction of free water.

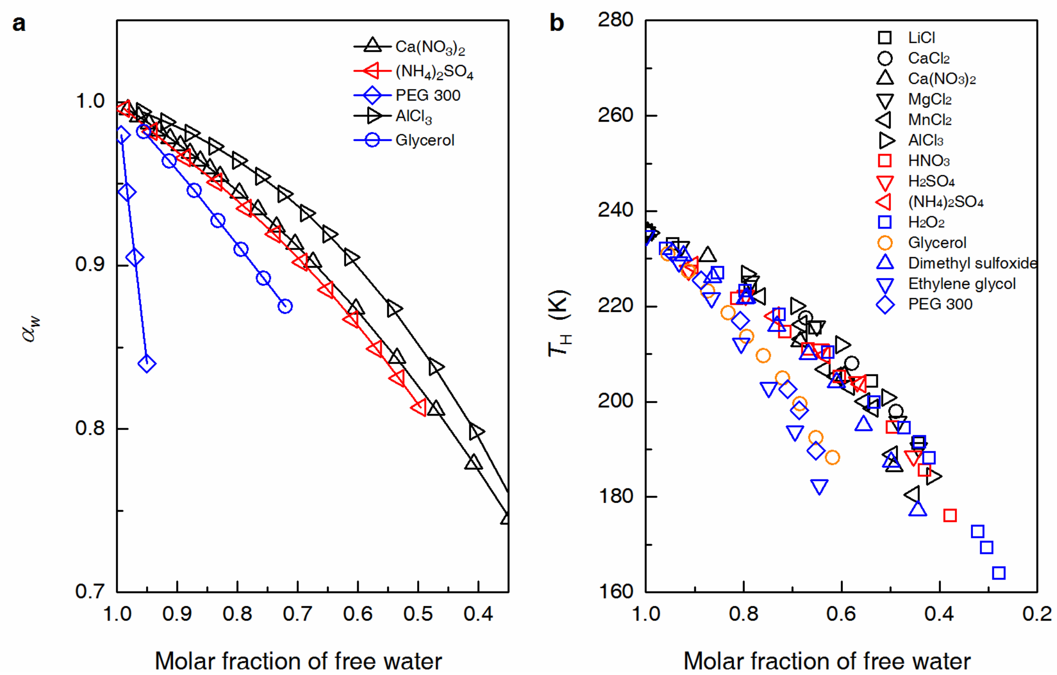


**Figure S14.** Water activity $a_{\mathrm{w}}$ (**a**) and homogeneous ice nucleation temperature $T_{\mathrm{H}}$ (**b**) versus molar fraction of free water in solution. The molar fraction of free water is given by $(n - n_{\mathrm{h}})/(n + v)$, where $n$ is the molar ratio of water to solute, $n_{\mathrm{h}}$ is the molar ratio of hydration water to solute, $v$ is the van't Hoff index of the solute. In ideal case, $v$ = 2 for LiCl, 3 for $Ca(NO_3)_2$, and 4 for $AlCl_3$, etc.

**Table S1.** Hydration number for various solutes determined from the $X^{h}_{aqu}$ value

a)

| Solute M | $X'_{aqu}$ | $n_h$ in $M \cdot n_h H_2O$ |
|---|---|---|
| LiCl | 0.72 | 6 |
| NaCl[a)] | 0.58 | 4.5 |
| $CaCl_2$ | 0.62 | 10 |
| $Ca(NO_3)_2$ | 0.43 | 7 |
| $MgCl_2$ | 0.70 | 12 |
| $ZnCl_2$ | 0.48 | 7 |
| $MgCl_2$+$ZnCl_2$ | 0.56 | 16.4 |
| $MnCl_2$ | 0.56 | 9 |
| $Mn(NO_3)_2$ | 0.54 | 11.7 |
| $CrCl_3$ | 0.66 | 17 |
| $Cr(NO_3)_3$ | 0.51 | 13.8 |
| $AlCl_3$ | 0.72 | 19 |
| $FeCl_3$ | 0.62 | 15 |
| $Fe(NO_3)_3$ | 0.54 | 15.8 |
| Glycerol | 0.23 | 1.5 |
| Ethylene glycol | 0.24 | 1.0 |
| Dimethyl sulfoxide | 0.41 | 3.0 |
| 1,2,4-butanetriol | 0.24 | 1.9 |
| PEG 300 | 0.31 | 6.8 |
| $H_2SO_4$[b,c)] | 0.56 | 6.9 |
| $HNO_3$[b)] | 0.54 | 4.1 |
| $H_2O_2$[c)] | 0.51 | 2 |
| $(NH_4)_2SO_4$[c)] | 0.34 | 3.6 |
| $HNO_3/H_2SO_4$($X_{H_2SO_4}$ = 0.03, 0.06) | 0.54 | |
| $H_2SO_4/HNO_3$($X_{HNO_3}$ = 0.07) | 0.56 | |

b) If $n_h$ = 4.5, $T_H$ of NaCl solutions measured at atmospheric pressure can also merge onto the master curve plotted in Figure 2b. In addition, it is known that $T_H$ = 207 K for pure water at 140 MPa and $T_H$ = 208 K for 4 mol. kg$^{-1}$ NaCl solution.[11] If eq. (2)

in the text describing the relationship between $P$ and $X_f$ is also valid for NaCl solutions, $n_h$ can be deduced and is also found to be about 4.5 (see Figure S11). This proposition is being verified by measuring emulsified NaCl solutions.

c) See Figure S2.

d) The concentration of freeze-concentrated phase for the $H_2O_2$, $H_2SO_4$, and $(NH4)_2SO_4$ solutions in this table was estimated in the following ways:

1) **$H_2O_2$ solution**. Bulk $H_2O_2$ solution crystallizes easily. Oguni et al. found that emulsified $H_2O_2$ solution can vitrify completely once its concentration is higher than that of $H_2O_2 \cdot 3H_2O$ (Figure3 from Ref.[7]), which corresponds to $X^{c}_{\mathrm{aqu}}$. The method developed in this study cannot be applied to determine the hydration number of $H_2O_2$ because of the independence of $T_g$ on solute concentration in the whole concentration range investigated. Dielectric study reveals that $H_2O_2 \cdot 2H_2O$ is stable enough against crystallization[7]. Accordingly, $X'_{\mathrm{aqu}}$= 0.51 for the $H_2O_2$ solution.

2) **$H_2SO_4$ solution.** For $H_2SO_4$ solution with a minor amount of $HNO_3$, the freeze-concentrated phase can completely vitrify, and its concentration remains constant, i.e. corresponds to $X^{c}_{\mathrm{aqu}}$= 0.56 (see Figure S2). Importantly, this value is insensitive to the amount of added $HNO_3$. Therefore, this value can be obtained by extrapolation for the pure $H_2SO_4$ solutions.

3) **$(NH_4)_2SO_4$ solution**. Homogeneous ice freezing in the aqueous solutions of $(NH_4)_2SO_4$ has been widely investigated by using different methods, e.g., Fourier-transform IR extinction spectroscopy, continuous flow diffusion, optical microscopy, and DSC measurement. Figure5 in Ref.[8] and Figure4 in Ref.[9] present a summation and

comparison among the published data.

The concentration of freeze-concentrated solution for the $(NH_4)_2SO_4$ solutions has recently been carefully analyzed by Bogdan and Loerting based on the measurement of freezing temperature of primary ice.[10] They proposed that the concentration of the freeze-concentrated solution, which freezes in the temperature range of 210~225 K, would center between ~0.61 and 0.66. Assuming that the solute concentration equals to 0.66 and consequently $X'_{\mathrm{aqu}}$= 0.34, then $T_{\mathrm{H}}$ for the $(NH_4)_2SO_4$ solutions can be well scaled onto a unique curve as a function of $X_{\mathrm{f}}$, see Figure 3b in the text.

**References**

1. Bogdan, A., Molina, M. J., Tenhu, H., Mayer, E. & Loerting, T. Formation of mixed-phase particles during the freezing of polar stratospheric ice clouds. *Nat. Chem.* **2**, 197-201 (2010)

2. Chang, H. Y. A., Koop, T., Molina, L. T & Molina, M. J. Phase transitions in emulsified $HNO_3/H_2O$ and $HNO_3/H_2SO_4/H_2O$ solutions. *J. Phys. Chem. A* **103**, 2673-2679 (1999).

3. Satoh, K. & Kanno, H. Anomalous crystallization behavior in the glass forming compositionregion of the $H_2O$-$HNO_3$ system. *Bull. Chem. Soc. Jpn.* **55**, 1645-1646 (1982).

4. Angell, C. A. Glass-forming composition regions and glass transition temperatures for aqueous electrolyte solutions. *J. Chem. Phys.* **52**, 1058 (1970).

5. Tielrooij, K. J., Garcia-Araez, N., Bonn, M. & Bakker, H. J. Cooperativity in ion

hydration. *Science* **328**, 1006-1009 (2010).

6. Stokes, R. H. & Robinson, R. A. Ionic hydration and activity in electrolyte solutions. *J. Am. Chem. Soc.* **70**, 1870-1878 (1948).

7. Minoguchi, A., Richert, R. & Angell, C. Dielectric studies deny existence of ultraviscous fragile water. *Phys. Rev. Lett.* **93**, 215703 (2004).

8. Larson, B. H. & Swanson, B. D. Experimental investigation of the homogeneous freezing of aqueous ammonium sulfate droplets. *J. Phys. Chem. A* **110**, 1907-1916 (2006).

9. Knopf, D. A. & Lopez, M. D. Homogeneous ice freezing temperatures and ice nucleation rates of aqueous ammonium sulfate and aqueous levoglucosan particles for relevant atmospheric conditions. *PCCP* **11**, 8056-8068 (2009).

10. Bogdan, A. & Loerting, T. Impact of substrate, aging, and size on the two freezing events of $(NH_4)_2SO_4/H_2O$ droplets. *J. Phys. Chem. C* **115**, 10682-10693 (2011).

11. Kanno, H. & Angell, C. A. Homogeneous nucleation and glass formation in aqueous alkali-halide solutions at high-pressures. *J. Phys. Chem.* **81**, 2639-2643 (1977).

12. Zobrist, B., Weers, U. & Koop, T. Ice nucleation in aqueous solutions of poly[ethylene glycol] with different molar mass. *J. Chem. Phys.* **118**, 10254 (2003).

13. Kanno, H., Soga, M. & Kajiwara, K. Linear relation between $T_H$ (homogeneous ice nucleation temperature) and $T_m$ (melting temperature) for aqueous solutions of sucrose, trehalose, and maltose. *Chem. Phys. Lett.* **443**, 280-283 (2007).

14. Mathlouthi, M., Luu, C., Meffroy-Biget, A. M. & Luu, D. V. Laser- Raman study of the solute-solvent interactions in aqueous solutions of D-fructose, D-glucose, and sucrose.

*Carbohyd. Res.* **81**, 213-223 (1980).

15. Mathlouthi, M., Cholli, A. L. & Koenig, J. L. Spectroscopic study of the structure of sucrose in the amorphous state and in aqueous solution. *Carbohyd. Res.* **147**, 1-9 (1986).
16. Mathlouthi, M. X-ray diffraction study of the molecular association in aquous solutions of D-fructose, D-glucose and sucrose. *Carbohyd. Res.* **91**, 113-123 (1981).
